# Anatomy of the right upper lobe revisited and clinical considerations in Chinese population

**DOI:** 10.1371/journal.pone.0242178

**Published:** 2020-11-25

**Authors:** Yan Chen, Ying Guo, Weidong Mi, Changsheng Zhang, Hong Wang, Dexu Zhao, Jiangbei Cao

**Affiliations:** Department of Anesthesiology, Anesthesia and Operation Center, The First Medical Center, Chinese PLA General Hospital, Beijing, China; University of Michigan, UNITED STATES

## Abstract

**Objective:**

The anatomy of the tracheobronchial tree differs among not only various races but also individual ethnic groups. Different lengths of the right mainstem bronchus (RMSB) had been described in previous publications. Since the differences in the anatomy of the RMSB and right upper lobe bronchus (RUB) may have clinical relevance when selecting devices, specifically, the right-side double lumen tube (R-DLT) for lung isolation, we revisited the anatomy of the right upper lobe in a large scale Chinese population.

**Methods:**

In this retrospective cohort study, we reviewed 2093 consecutive adult patients undergoing thoracic computed tomography (CT) scans from data base in our hospital. Demographic characteristics were collected. The lengths, internal diameters, and angles of the RMSB and RUB were measured using reconstructive CT images. The correlations between the demographic variables and the RMSB length and diameters were also analyzed.

**Results:**

The incidence of the aberrant RUB originated equal or above the tracheal carina was 8.1‰. 52.3% of the patients had a length of RMSB less than 23 mm, and the incidence of RMSB length <23 mm in women was significantly higher than that in men (63.5% vs. 42.8%, *p =* 0.000). The right bronchial length (RBL) was less than 10 mm in 21% of cases (17.8% in men and 24.8% in women, respectively, *p =* 0.000). Both the RMSB lengths and diameters had poor correlations with the heights in either male or female patients.

**Conclusion:**

A much higher incidence of a shortened RMSB potentially make placement of a R-DLT more difficult in Chinese population. Both the lengths and diameters of the RMSB cannot be predicted by the height. Preoperative thoracic CT scan for each patient helps optimizing the selection of a lung isolation device, and the importance of an evaluation of the CT scans preoperatively by the anesthesiologists should be emphasized.

## Introduction

The anatomy of the tracheobronchial tree differs among various races, and even differs from different ethnic groups [1]. The differences in the bronchial anatomy mostly attributed to the racial discrepancy and congenital anatomic anomaly. Approximately 10% of patients presented some form of tracheobronchial anomaly, and most of the major anomalies which were in the area of the lobar or primary branchings were noted on the right [2]. The incidence of the aberrant right upper lobe bronchus (RUB) originated equal or above the tracheal carina had been reported as 0.1%-2% [3–8]. Both the right mainstem bronchial anatomy and ectopic variation of the RUB may have clinical relevance to surgical procedures and airway management, therefore, accurate anatomic information of the right mainstem bronchus (RMSB) and RUB helps optimizing surgical protocol and airway management as well as improving medical equipment. For anesthesiologists, knowledge of the right bronchial patterns including some anatomic variations is very important to perform safe and appropriate placement of a right-side double-lumen endobronchial tube (R-DLT).

Different mean RMSB lengths ranged from 12 to 25.5 mm had been described in previous publications [9–13] (Table 1). In these studies, aside from different methods, such as in vivo fiberoptic bronchoscopy, autopsy study and cast study as well as computed tomography (CT) scan, were used to measure the RMSB length, the definition of the RMSB length were ambiguous. In some studies, the distance between the tracheal carina and the proximal margin of the upper lobe bronchial orifice was defined as the right bronchial length (RBL) [9–11], however, other researchers defined the RMSB length as the distance between the tracheal carina and the distal margin of the upper lobe bronchial orifice [12, 13]. Since accurate anatomy of the RMSB and RUB relates to the selecting of devices, specifically, the R-DLT for lung isolation, definite RMSB length need to be further discussed.

**Table 1:** Measurements of the right mainstem bronchial length in previous publications.

Publications (year)	Cases (male/female)	RBL/RMSBL (mm)			Measurement method
		Male	Female	Combined	
Benumof et al.(1987)	69(36/33)	19±8	14±7	17±8	In vivo FB
	42(32/10)	19±6	16±4	18±6	Autopsy
	55	-	-	18±7	Lung cast
Youngberg (2000)	-	-	-	22	-
Hagihira et al.(2008)	80(55/25)	13.5	11.7	-	Plain X-ray
Kim et al.(2013)	76(50/26)	-	-	12	CT scan
Mi et al. (2015)	2107(1134/964)	14.1±4.5	12.9±4.0	13.6±4.3	CT scan
Bussieres et al. (2019)	106(52/54)	-	-	25.5±4.7	CT scan

Values are shown as mean ± SD. RBL: right bronchial length; RMSBL: right mainstem bronchial length; RUBD: right upper bronchus internal diameter; FB: fiberoptic bronchoscopy; CT: computed tomography.

In recent years, CT-guided bronchial width measurement has been used to predict the appropriate DLT size increasingly, which is superior to conventional method, such as height, age, and chest X-ray [14]. A recent study involving 106 patients had provided a better understanding of the variable anatomy of the right bronchial tree by using thoracic CT imaging [13]. In 2015, our team had published a study in regard to the precise measurements of the tracheobronchial tree using CT scans in Chinese population, which focused on the entire tracheobronchial distribution, and found that there was a large individual variation in the length of the RMSB [11]. Here, according to the Kim’s method [12], we redefined the length of RMSB, and revisited the anatomic features of the RMSB and RUB using CT scans in a large scale study. We also analyzed the correlations between the right bronchial parameters and demographic characteristics, which aimed to propose useful suggestions regarding evaluation of the airway and selection of the devices for lung isolation.

## Material and methods

We reviewed 2690 consecutive patients, who underwent radiologic thoracic CT scans from April 2011 to May 2013 in the first medical center of the Chinese PLA General Hospital. This retrospective cohort study was approved by the Ethics Committee of the Chinese People’s Liberation Army General Hospital (approval number: S2019-311-02). All patients provided informed written consent to have data from their medical records used in research, and all data were fully anonymized before we accessed them. Exclusion criteria includes non-Chinese, younger than 18 or older than 90-year-old, unable to cooperation for hearing impairment, psychiatric disorders or intellectual disabilities, prior diagnosis of compulsive position, musculoskeletal deformity, or thoracic injury, and a history of tracheobronchial intubation or surgery. The qualified 2093 patients were enrolled in this study. Demographic information (including age, gender, height, and weight) of the patients were also collected.

All patients received thoracic CT scans using a SOMATOM Emotion 16 scanner (Simens Healthcare, Forchheim, Germany). The scanned CT images were stored in DICOM format in the local area network server of the Hospital. CT images for each patient were reconstructed and measured using the image measurement software UniWeb Viewer 6.1.1152 (EBM Technologies, Inc; Beijing, China). A lung window image sequence with a slice thickness of 1.5 mm was used for the measurements.

**Fig 1:**
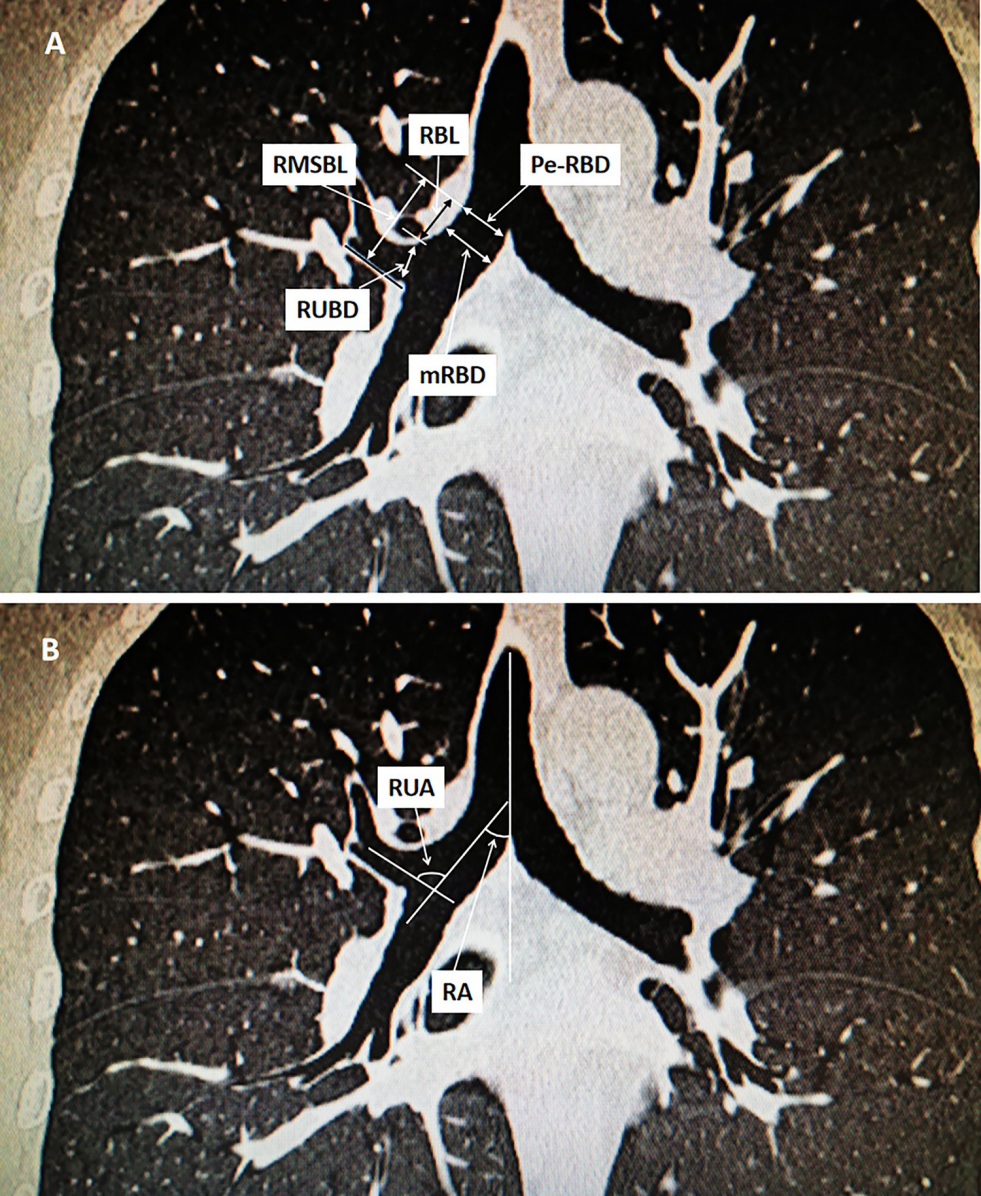
Illustration of the different right bronchial measurements and angles using reconstructive CT images. A. RMSBL: right mainstem bronchus length; RBL: right bronchus length; Pe-RBD: proximal end right main bronchus internal diameter; mRBD: mid-right main bronchus internal diameter; RUBD: right upper bronchus internal diameter. B. RA: right bronchial angle; RUA: right upper bronchial angle.

The different right bronchial measurements and angles were illustrated in Fig 1. The distance between the tracheal carina and the distal margin of the right upper lobe orifice was measured as the RMSB length (RMSBL). The right bronchial length (RBL) was the distance between the tracheal carina and the proximal margin of the right upper lobe orifice. Pe-RBD (proximal end right main bronchus internal diameter), mRBD (mid-right main bronchus internal diameter), and RUBD (right upper bronchus internal diameter) were also measured. The right bronchial angle (RA) was measured in degrees from the tracheal axis, and the right upper bronchial angle (RUA) was measured in degrees from the right bronchial axis. Each data point was measured three times in the presence of a radiologist and an anesthesiologist, and the mean of three measurements as the measured values. For patients who had the CT scans of extremely shortened RBL or RUB located equal or above the tracheal carina on two-dimentional images, three-dimentional imaging using three-dimention reconstruction software (Jingzhentech, Beijing, China) was used to confirm the aberrant RUB.

Statistical analysis was performed using SPSS 17.0 software (SPSS Inc, Chicago, IL). All Data were presented as median and interquatile range (IQR), and percentage as appropriate. The gender-related differences in the demographic variables and bronchial tree parameters were compared using the nonparametric test for independent samples. Categoric variables were compared using the X^2^ test. Bivariate correlations were used to analyze the relationship between the bronchial parameters and demographic variables. A *p* value less than 0.05 is considered statistically significant.

## Results

2093 patients undergoing thoracic CT scans, including 1136 male and 957 female, were enrolled in the study. These patients include outpatients and inpatients, and there were 68 patients who underwent lung surgery, including 35 patients receiving R-DLT intubation for left lung surgery. The use of R-DLT was replaced by a EZ-blocker in only one patient with RBL < 8 mm. In another patient with RBL < 8 mm, an R-DLT was repositioned from the right main bronchus into the left main bronchus. Demographic variables for all patients were compared in Table 2. The patients ranged in age from 18 to 89 years old, height from 145 to 192 cm, and weight from 40 to 115 kg. No significant differences was seen in the age between the male and female patients [52 (41, 60) yr vs. 51(42, 59) yr, *p =* 0.582]. However, there were significant differences in the height and weight between the male and female patients [172 (168, 175) cm vs. 160 (157, 164) cm, *p* = 0.000 and 70 (63, 77) kg vs. 60 (53, 65) kg, *p* = 0.000, respectively].

**Table 2:** Demographic variables of the patients.

Variables	Male (n = 1124)	Female (n = 952)	*P* value
Age (yr)	52(41,60)	51(42,59)	0.582
Height (cm)	172(168,175)	160(157,164)	0.000
Weight (kg)	70(63,77)	60(53,65)	0.000

Values are shown as median and interquatile range (IQR).

**Fig 2:**
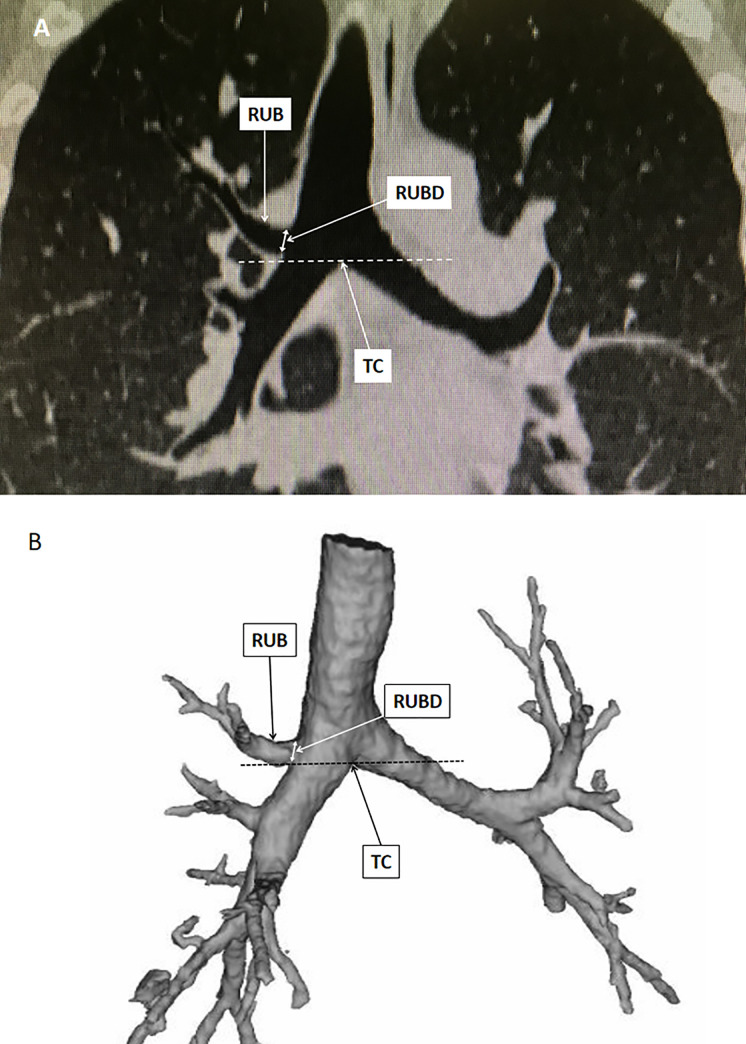
Identify aberrant right upper bronchus using two-dimentional and three-dimentional reconstruction techniques in a individual patient, respectively. A. two-dimentional reconstruction image; B. three-dimentional reconstruction image. (RUB: right upper bronchus; RUBD: right upper bronchus internal diameter; TC: trachea carina).

**Fig 3:**
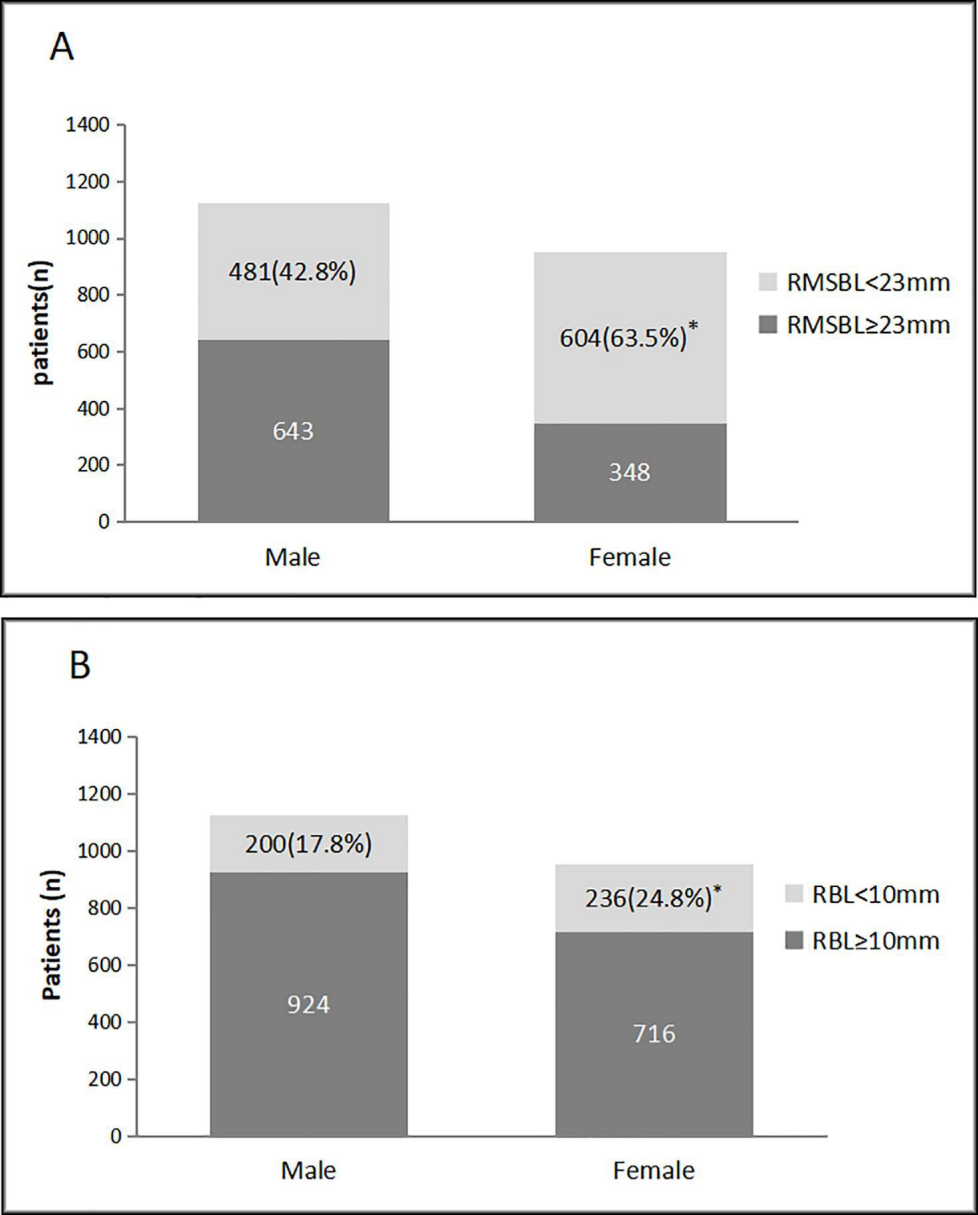
Distribution of the right mainstem bronchus length in the patients (n = 2076). A. The length of the right mainstem bronchus (RMSB) is less than 23 mm in 52.3% of patients. The incidence of RMSB < 23 mm in women was significantly higher than that in men (63.5% vs. 42.8%, *p = 0.000). B. The right bronchus length (RBL) is less than 10 mm in 21% of patients. The incidence of the RBL <10 mm in women was significantly higher than that in men (24.8% vs. 17.8%, *P = 0.000).

There were 17 patients (12 male, 5 female) with the RUB originated equal or above the tracheal carina. A representative two-dimentional and three-dimentional images of the aberrant RUB in a single patient were showed in Fig 2. With the exception of the patients with aberrant RUB, the median (IQR) length of the RMSB in all patients was 22.7 (19.6, 26.2) mm (range: 10.2–42.1 mm). The median values of the RMSB length (RMSBL), RBL, Pe-RBD, mRBD, and RUBD for the male patients were significantly higher than those for the female patients [23.7 (20.7, 27.0) mm vs. 21.5 (18.6, 24.9) mm, *p* = 0.000; 13.8 (11.0, 17.0) mm vs. 12.6 (10.0, 15.5) mm, *p* = 0.000; 14.1 (12.8, 15.4) mm vs. 12.3 (11.1, 13.4) mm, *p* = 0.000;14.1 (12.7, 15.5) mm vs. 12.1 (10.9, 13.4) mm and 9.7 (8.6, 11.0) mm vs. 8.7 (7.8, 9.9) mm, *p* = 0.000, respectively] (Table 3). The distribution of the RMSBL and the RBL in the patients was showed in Fig 3. 52.3% of the patients had a RMSBL less than 23 mm. The incidence of RMSBL <23 mm in women was significantly higher than that in men (63.5% vs. 42.8%, *p* = 0.000). The RBL is less than 10 mm in 21% of patients, and the incidence of the RBL <10 mm was 17.8% in men and 24.8% in women (*p* = 0.000). The median (IQR) value of RA in female patients was larger than that in the male patients [35.8 (30.4, 40.5) degrees vs. 33.5 (28.5, 38.8) degrees, *P* = 0.000]. However, no significant gender differences was found in the RUA [73.3 (63.0, 85.0) degrees in male vs. 73.5 (62.7, 85.3) degrees in female, *p =* 0.792] (Table 3).

**Table 3:** Measurements of the right bronchial variables (n = 2076).

Variables	Male	Female	Combined	*P* value
RMSBL (mm)	23.7(20.7,27.0)	21.5(18.6,24.9)	22.7(19.6,26.2)	0.000
RBL (mm)	13.8(11.0,17.0)	12.6(10.0,15.5)	13.3(10.5,16.3)	0.000
Pe-RBD (mm)	14.1(12.8,15.4)	12.3(11.1,13.4)	13.2(11.8,14.6)	0.000
mRBD (mm)	14.1(12.7,15.5)	12.1(10.9,13.4)	13.1(11.8,14.7)	0.000
RUBD (mm)	9.7(8.6,11.0)	8.7(7.8,9.9)	9.3(8.0,10.6)	0.000
Angles (in degrees from tracheal or bronchial axis)				
RA	33.5(28.5,38.8)	35.8(30.4,40.5)	34.3(29.2,39.5)	0.000
RUA	73.3(63.0,85.0)	73.5(62.7,85.3)	73.3(62.9,85.1)	0.792

Values are shown as median and interquatile range (IQR). RMSBL: right mainstem bronchus length; RBL: right bronchus length; Pe-RBD: proximal end right main bronchus internal diameter; mRBD: mid-right main bronchus internal diameter; RUBD: right upper bronchus internal diameter; RA: right bronchial angle; RUA: right upper bronchial angle.

**Fig 4:**
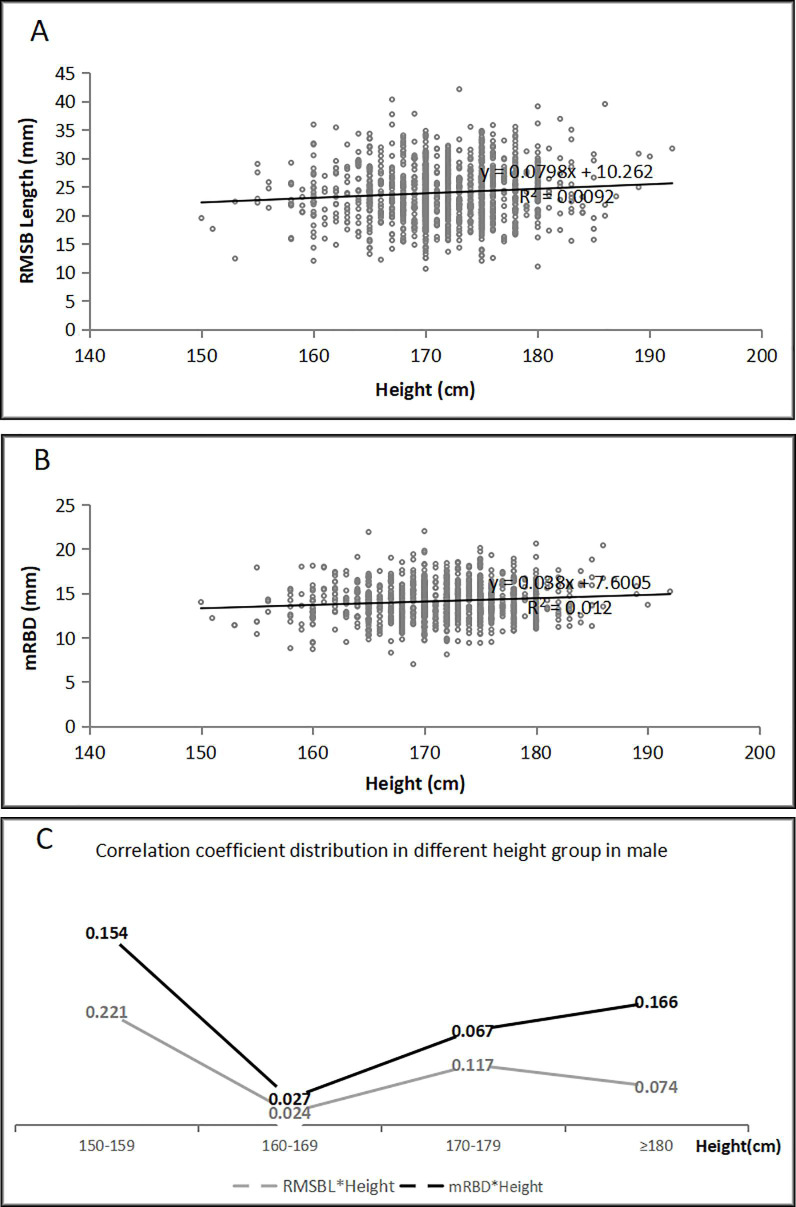
The correlations between the height and the RMSB length or diameter in the male patients (n = 1124). A. The RMSB length was poorly correlated with the height (r = 0.096, *p* = 0.001). B. The mRBD was poorly correlated with the height (r = 0.110, *p* = 0.000). C. The correlation coefficient distribution in different height group. (RMSB: right mainstem bronchus; mRBD: mid-right main bronchus internal diameter; RMSBL: right mainstem bronchus length).

**Fig 5:**
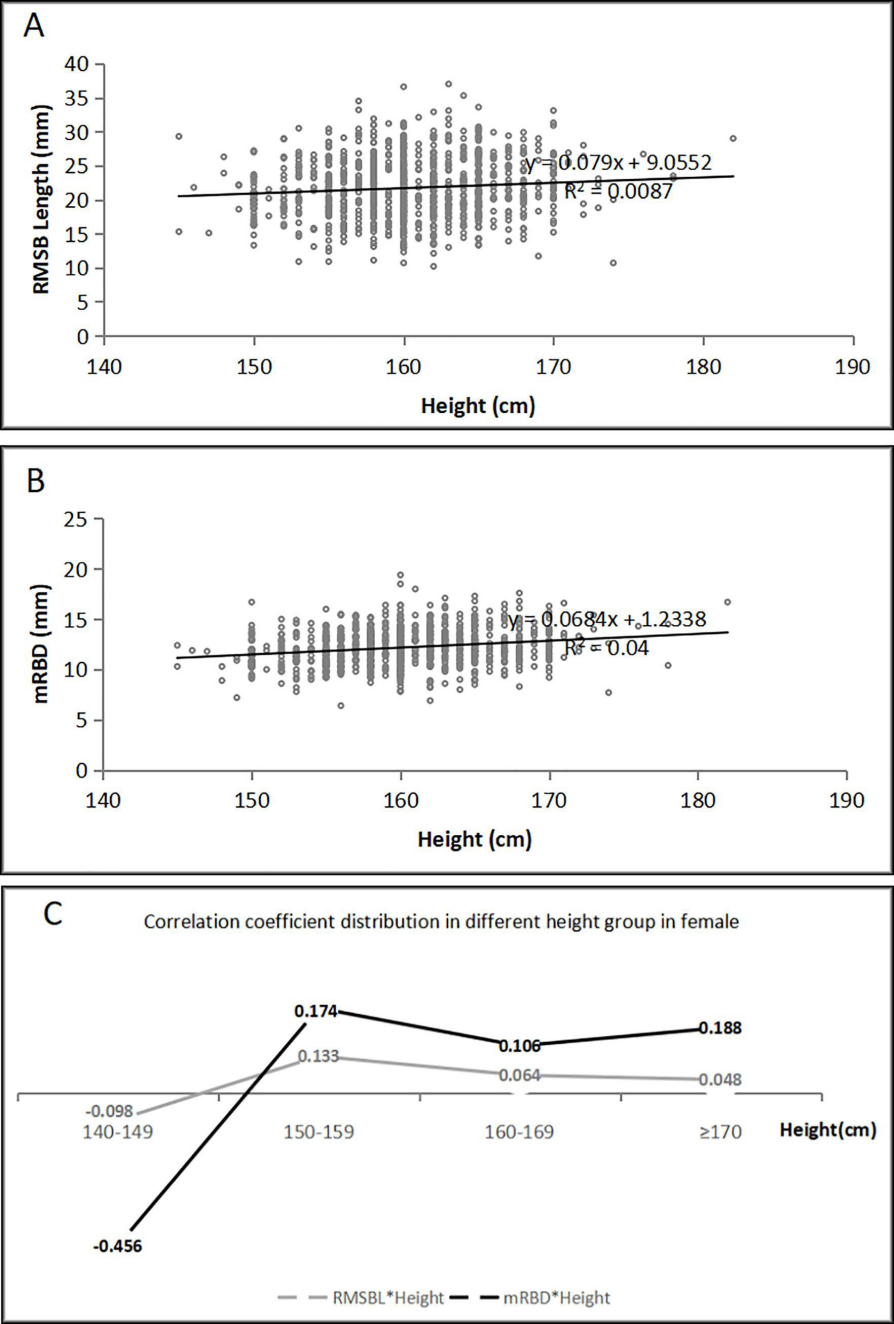
The correlations between the height and the RMSB length or diameter in the female patients (n = 952). A. The RMSB length was poorly correlated with the height (r = 0.093, *p* = 0.004). B. The mRBD was poorly correlated with the height (r = 0.200, *p* = 0.000). C. The correlation coefficient distribution in different height group. (RMSB: right mainstem bronchus; mRBD: mid-right main bronchus internal diameter; RMSBL: right mainstem bronchus length).

The RMSB length had poor correlations with the age, height, Pe-RBD and mRBD [The correlation coefficient (r) = 0.086 (*p* = 0.004), r = 0.096 (*p* = 0,001), r = 0.090 (*p* = 0.003) and r = 0.078 (*p* = 0.009) for men and r = 0.105 (*p* = 0.001), r = 0.093 (*p* = 0.004), r = 0.119 (*p* = 0.000) and r = 0.110 (*p* = 0.001) for women, respectively], however, it had no significant correlation with the weight (r = 0.012, *p* = 0.691 for men and r = 0.001, *p* = 0.976 for women). Both the Pe-RBD and mRBD were poorly related to the height (r = 0.127, *p* = 0.000 and r = 0.110, *p* = 0.000 for men and r = 0.217, *p* = 0.000 and r = 0.200, *p* = 0.000 for women, respectively). The mRBD had poor correlation with the age in men (r = 0.074, *p* = 0.013) but not in women (r = 0.042, *p* = 0.190), and there were no significant correlations between the Pe-RBD and the age (r = 0.015, *p* = 0.606 for men and r = 0.006, *p* = 0.856 for women). The relationships between the RMSBL, mRBD and the age, height were presented in Fig 4 for men and Fig 5 for women. The RUBD was poorly related to the height (r = 0.113, *p* = 0.000 for men and r = 0.120, *p* = 0.000 for women), and it also was poorly related to the age in women (r = 0.070, *p* = 0.030) but not in men (r = 0.017, *p* = 0.576). There were no significant correlations between the RUBD and the weight (r = -0.013, *p* = 0.669 for men and r = -0.033, *p* = 0.308 for women).

## Discussion

### Higher rate of a shortened RMSB in the Chinese population

The variable anatomy of the RMSB and RUB is the main reason of the misalignment of the lateral orifice of the R-DLT. From the Kim’s study [12], the distance from the proximal end of the endobronchial cuff to the distal edge of the ventilation slot of the Broncho-Cash DLT (Mallinckdrot Medical Ltd., Athlone, Ireland) is 23 mm, a measure which predicts R-DLT malpositioning. The results of our study showed a much higher rate (52.3%) of RMSB length less than 23 mm (63.5% in women, 42.8% in men) in our patients. This percentage is comparable with that (50%) in Korea patients, and differs from that (25%) in Canadian patients [4, 13]. The Kim’s study had a very small number of cases, inversely, our study included the greatest number of patients (n = 2093) of all relevant studies except for our own previous study, as listed in Table 1, so the percentage is convincing. In addition, our hospital provides medical service open to the patients from all over the country, and the higher incidence of a shortened RMSB in Chinese population may alert anesthesiologists considering the possibility of R-DLT malpositioning.

The RBL measured in our study differs from those in other countries [2, 15, 16]. The rate (8.1‰) of the RUB originated from the trachea was within ranges reported by previous studies [3–7]. The length of the Broncho-Cath right cuff was 10 mm for all size, and the average margin of safety in RBL was 8 mm. The RBL are shorter than 10 mm in 21% of our patients (17.8% in men and 24.8% in women), and a standard R-DLT wound not be feasible for these patients. Our percentage is similar to that (20%) in Japanese patients, and higher than that (11%) in American patients [9, 19]. Since the Broncho-Cath DLT represents the majority of the R-DLT used in China, considering the higher incidence of the RMSBL < 23 mm or the RBL < 10 mm, more patients (especially female patients) of our country might be confronted with the malpositioning of an R-DLT, which possibly resulting in right upper lobe obstruction and inadequate ventilation. Additionally, If the tube length exceed the length of the right bronchus by 10 mm, airway trauma and rupture of the membranous part of the trachea may occur [17]. Therefore, it is essential to evaluate preoperative CT images to determine which lung isolation device is appropriate. The percentage of R-DLT use is approximately 30% - 40% in patients undergoing lung surgery in our hospital in recent years. If the RUB locates equal or above the trachea carina or the RBL length is < 10 mm according to preoperative CT images, the use of R-DLT would be replaced by other methods such as EZ-blocker and left-side DLT (L-DLT). If the R-DLT is applicable, the tube should be aided by a flexible fiberoptic bronchoscope to allow a better positioning. When unexpected RUB occlusion and difficult repositioning occur during surgery, repositioning an R-DLT from the right main bronchus into the left main bronchus may be an efficient method [18].

The average margin of safety with Rusch DLT is very small (1–4 mm) [9], and the right upper lobe obstruction may occur in more cases in patients with a shortened RMSB. Hagihira S et al. [19] reported a newly modified R-DLT (the Cliny rDLT) with two ventilation slots being used in two patients with very short RMSB. Bussieres et al. [20] proposed a Broncho-Cath tube modification that includes an enlarged lateral orifice to improve positioning of the R-DLT. However, no modified R-DLT is commercially available in China. Our results may promote the development of a newly designed R-DLT which is suitable for Chinese people, but now, the EZ-blocker may be a better choice for patients with short RMSB.

### Poor correlations between the RMSB length or diameters and the heights

Although a positive linear correlation between the heights of the patients and the lengths of the RMSB was observed (r = 0.39), the r value is of limited clinical significance due to a small number of cases [2]. Based on a large number of cases, our study revealed that the heights are poorly related to the RMSB lengths (r = 0.096 in men and r = 0.093 in women), and its predictive value is extremely limited for individual patients. Our study population represents most of the adult and elderly people (the age ranged from 18–89 year and the height ranged from 145–192 cm), therefore, it is unreliable for most of the patients to using the height to predict the RMSB length. If the height of a patient is out of this range, the correlation between the right bronchial parameters and the height should be reconsidered. Additionally, the RMSB diameters are poorly related to the heights, and the RMSB lengths also are poorly related to the diameters of the RMSB. Therefore, preoperative CT evaluation is very important for us to select a appropriate R-DLT size. If difficult in R-DLT placement according to preoperative CT images, our suggestion for lung isolation is as follows: (1) If surgical procedure wound not be affected, L-DLT would be better choice; (2) If the use of L-DLT affect surgical procedure, EZ-blocker would be used as a device for lung isolation; (3) For a long and thin RMSB, placement of a smaller size tube may reduce membrane injury; (4) A flexible fiberoptic bronchoscope should be regularly used to guide the positioning of a tube in order to ensure adequate ventilation and reduce the airway injury.

Our study also has some limitations. First, the threshold value of RMSBL < 23 mm or RBL <10 mm predicting R-DLT malpositioning arises from the specifications of the Broncho-Cath DLT only, and thus, our results may not be applicable with other types of lung isolation devices. Second, this is a retrospective investigation with very small number of patients who underwent lung surgery, therefore, the detailed data on whether there was difficulty in placing the R-DLT in patients with a RMSBL< 23 mm or RBL < 10 mm are unavailable. A prospective, larger scale of study with more patients undergoing lung surgery is needed in the future.

In summary, our results revealed the unique aspects of the right upper lobe bronchial anatomy among Chinese population. A much higher incidence of a shortened RMSB of the patients indicates more difficulties in placement of a R-DLT. Preoperative thoracic CT scan for each patient helps optimizing the selection of a lung isolation device, and the importance of an evaluation of the CT scans preoperatively by the anesthesiologists should be emphasized. Further, when two-dimentional radiological imaging is not sufficient in detecting some bronchial anomalies, three-dimentional reconstruction techniques are needed to confirm the anatomic anomaly.
